# Insight dimensions and cognitive function in psychosis: a longitudinal study

**DOI:** 10.1186/1471-244X-6-26

**Published:** 2006-05-31

**Authors:** Manuel J Cuesta, Victor Peralta, Amalia Zarzuela, Maria Zandio

**Affiliations:** 1 Psychiatric Unit of "Virgen del Camino" Hospital. E-31008 Pamplona, Spain

## Abstract

**Background:**

It has been reported that lack of insight is significantly associated with cognitive disturbance in schizophrenia. This study examines the longitudinal relationships between insight dimensions and cognitive performance in psychosis.

**Methods:**

Participants were 75 consecutively admitted inpatients with schizophrenia, affective disorder with psychotic symptoms or schizoaffective disorder. Assessments were conducted at two time points during the study: at the time of hospital discharge after an acute psychotic episode and at a follow-up time that occurred more than 6 months after discharge. A multidimensional approach of insight was chosen and three instruments for its assessment were used: the Scale to Assess Unawareness of Mental Disorder (SUMD), three items concerning insight on the Assessment and Documentation in Psychopathology (AMDP) system and the Insight and Treatment Attitudes Questionnaire. The neuropsychological battery included a wide range of tests that assessed global cognitive function, attention, memory, and executive functions.

**Results:**

After conducting adequate statistical correction to avoid Type I bias, insight dimensions and cognitive performance were not found to be significantly associated at cross-sectional and longitudinal assessments. In addition, baseline cognitive performance did not explain changes in insight dimensions at follow-up. Similar results were found in the subset of patients with schizophrenia (*n *= 37). The possibility of a Type II error might have increased due to sample attrition at follow-up.

**Conclusion:**

These results suggest that lack of insight dimensions and cognitive functioning may be unrelated phenomena in psychosis.

## Background

Psychosis is expressed through a wide range of psychopathological phenomena and different outcomes. The neurobiological underpinnings of psychosis remain unknown despite extensive research in the last century. The clinical heterogeneity within psychosis has led to a debate concerning what the 'core' manifestations of psychosis are, which is still progressing [1]. However, it is widely acknowledged that lack of insight is the most prevalent symptom in psychosis [2] and it has a notable influence on cooperation with treatment and clinical outcome in psychotic patients [3].

Insight involves different underlying phenomena and now includes multidimensionality as an attribute [4]. Two multidimensional models are widely accepted. David [5] hypothetized that insight comprises three dimensions: recognition that one has a mental illness, the ability to relabel unusual mental events as pathological, and adherence with treatment. These insight dimensions are overlapping, dimensional and dynamic phenomena, which allow for particular variations along the course of the illness. This model holds face validity and has also been used by our group and others [6]. The other multidimensional model, proposed by Amador et al [4], distinguishes between the two main components of insight: unawareness of illness and incorrect attribution of deficit or consequence of illness. Provided that insight is a dynamic symptom domain and may change along the course of the illness, Amador et al developed an assessment scale that allows for current and retrospective assessments of these two main components of insight.

Explanations of the causes of lack of insight are purely hypothetical and pending clarification. Psychological processes [7,8], neurocognitive mechanisms [4] and psychopathological explanations have been proposed as explanations [9]. Two methods for exploring the hypothetical neurocognitive dysfunction associated with lack of insight have been used: neuroimaging procedures and neuropsychological assessment. Inconsistent findings have been reported by nine neuroimaging studies that focused on insight. Four out of the nine studies reported significant associations between lack of insight and either ventricular enlargement [10], smaller brain and intracraneal volumes [11], frontal lobe atrophy [12], bilateral volume reductions in middle frontal gyrus, gyrus rectus, and left anterior cingulate gyrus [13] or paralimbic structures [14]. However, these results should be taken as inconclusive due to severe limitations in the studies that reported positive findings. These limitations included the absence of specific instruments for the assessment of insight [10] and the inclusion of small samples [11,12]. In addition, many of these studies were performed with patients with chronic schizophrenia, making it difficult to disentangle the effects of illness chronicity and exposure to antipsychotic drugs. To overcome this limitation, two studies examined the relationship between insight in first-episode psychotic patients and specific brain regions, such as the cingulated gyrus and dorsolateral prefrontal cortex, respectively [15,16]. In addition, significant correlations were not found between cerebral ventricular enlargement and insight in a large set of individuals with recent onset psychosis [17], as well as in another recently published study from the same group [18].

Conflicting findings have emerged concerning the relationship between insight and neuropsychological performance in psychosis. Although several methodological limitations have hampered the results, 37 studies (33 cross-sectional and four longitudinal studies) have been carried out. Out of the 33 cross-sectional studies, 22 showed significant relationships with executive measures [7,8,18-30] or other neuropsychological tests [31-36]. In addition, significant relationships between insight and neuropsychological [9,37-44] or intellectual performance [11,45] were not found in one-third of the 33 cross-sectional studies. In regards to longitudinal studies, three out of the four studies did not show significant associations between insight and cognitive performance [17,46,47]. However, executive and memory domains were associated with lack of insight in one study [48] and positive attitudes towards medication were associated with better performance on tests of verbal working memory in a forensic sample of patients with schizophrenia [49].

The variability in these findings may be accounted for by the different instruments used to assess insight and cognitive functioning, design limitations (i.e., cross-sectional design), or a lack of statistical power in the studies. The present study was conducted to examine if cognitive dysfunction can predict the degree of insight into illness in patients suffering from psychosis. A dimensional approach and a longitudinal design were used. Specifically, our study was developed from the hypothesis that lack of insight is not determined by a neuropsychological disturbance. The objectives were to ascertain: whether cross-sectional neuropsychological disturbance is associated with insight dimensions at the time of hospital discharge after a psychotic episode and after a long period of stabilization and if baseline neuropsychological performance predicts disturbances on insight dimensions at follow-up.

## Method

The study design was longitudinal and involved assessments at two different time points. The assessments measured multidimensional aspects of insight, psychopathological status and neuropsychological performance in a group of patients suffering from psychosis. This report presents a brief description of the study's methodology, as a description of the sample and psychopathological and insight assessment procedures have been reported elsewhere [50].

### Patients

The study group consisted of 75 patients who were consecutively admitted to a psychiatric unit due to an acute psychotic episode. The patients were assessed after the remission of the acute episode and six months to two years after discharge from the hospital. The second assessment occurred at a time when the patient was experiencing a phase of clinical stabilization (*n *= 56). Patients met DSM-IV [51] criteria for schizophrenia (*n *= 37), affective disorder with psychotic symptoms (*n *= 27) and schizoaffective disorder (*n *= 11). The Comprehensive Assessment Schedule History (CASH) [52] was used as a semistructured interview derive each patient's diagnosis and to assess psychopathology at baseline and follow-up. Inter-rater reliabilities for the positive and negative symptom scales have been reported as good to excellent [53]. The psychopathological, depressive and manic dimensions from Liddle's three-syndrome model [54] were computed. Clinicians completed the Clinical Global Impression scale (CGI) [55]. The CGI is a 7-point scale ranging from 1(*very much improved*) to 7(*very much worse*). This scale was completed during the baseline and follow-up assessments periods of this study.

There were 19 patients that dropped out of the study and were not available for follow-up assessment. Our research program and its included protocols were approved by the Ethic Committee of our Hospital. Participants gave their informed consent to collaborate in the assessment procedures. Patients with a history of organic central nervous system disorder, drug or alcohol abuse in the past year or mental retardation were excluded.

### Procedure and assessment measures

The assessment of insight utilized three instruments: (a) the Scale to assess Unawareness of Mental Disorder (SUMD) [56], (b) the Insight and Treatment Attitudes Questionnaire (ITAQ) [57], and (c) three insight items from the Manual for the Assessment and Documentation in Psychopathology (AMDP) [58]. The AMDP items were lack of feeling ill, lack of insight and uncooperativeness. Insight assessment was conducted in two sessions: the AMDP insight items and the SUMD were administered during the first session and the ITAQ was administered during the second session. The SUMD assessments were conducted by one of the researchers (MJC). The AMDP items were scored by MJC & VPM. The third researcher (AZ) scored the ITAQ. The authors participated in a pilot study in order to reach a consensus on ratings that were obtained using the three insight instruments. The procedure for this pilot study involved the authors completing independent ratings of interviews that were conducted with fifteen patients who were consecutively admitted to the hospital with a psychotic episode. This procedure was followed by a discussion about each patient in order to reach consensus ratings. High inter-rater reliability between the two raters for AMDP-insight items has been previously reported [9]. Each evaluator was blind to the measures of the other researchers. A description of the three instruments has been reported elsewhere [50].

### Neuropsychological assessment

A neuropsychological battery was selected to provide a wide assessment of cognitive functions. These instruments measured cognitive functions that are commonly disturbed in schizophrenia, such as attention, memory and executive functions. The Information test of the WAIS was used to assess global [59]. Immediate and delayed verbal memory, and verbal fluency were assessed by means of subtests of a Spanish-adapted neuropsychological battery [60]. The immediate and delayed Verbal Memory tasks of our battery were very similar to the Logical Memory tests of the Wechsler Memory Scale. The Verbal Fluency test consisted of the recording of the number of animals in 1 minute. Attention and executive function were assessed with two tests: the form B of the Trail Making Test [61] and the Stroop Color Word Test [62]. Finally, the Wisconsin Card Sorting Test (WCST) [63] was also used as a measure of executive function. Baseline insight and cognitive assessments took place after remission of the acute episode, during the days just before discharge (mean admission duration 3.67 ± 3.1 weeks).

### Statistical analysis

Initially, inspection of variables was carried out and those variables non-normally distributed were transformed. Pearson correlation analyses between cognitive and insight measures were carried out at baseline and follow-up assessments. Bonferroni inequality correction was chosen to account for a high number of statistical tests [64]. The potential confounding effect of length of illness and illness severity over cognition and insight was tested by means of 'partial correlation' analyses.

As a complementary analytical methodology used to avoid conducting a large number of statistical procedures and to analyze insight dimensions, the data was summarized by means of two separated 'principal component factor analyses' of baseline insight and of neuropsychological variables. Oblique rotation was chosen to avoid symptom overlapping on factors and to extract meaningful factors of cognitive variables. In order to match these baseline factors with the same factors at the follow-up assessment, the same variables were used in two 'factor analyses' that were carried out at follow-up. The number of resulting factors was fixed 'a priori' to the same number of factors of respective baseline assessments.

Longitudinal relationships between psychopathology and insight were analyzed through multiple regression analyses. Specifically, we assessed whether baseline cognitive dimensions predicted change in insight at follow-up. Prediction estimates were calculated through a stepwise multiple regression. The variables were entered into the model at a probability of *F *= 0.05, and were removed at *F *= 0.1. Statistical analyses were performed using Statistical Package for the Social Sciences (SPSS Inc, Chicago).

## Results

Table 1 displays the descriptive results of the demographic, insight and neuropsychological variables. Attrition bias (25%) during the study was examined through comparisons of basal insight and basal cognitive dimensions between refusing patients and those patients who remained in the study. Patients who refused showed lower educational background than patients who remained in the study (*t *= 2.44, *p *≤ 0.01). No significant statistical differences between these two groups of patients on insight or neuropsychological measures were found. A trend towards significant differences was found between these two groups on the lack of feeling ill on the AMDP, however this result did not reach statistical significance after a correction for the number of tests that were performed (*t *= -.2.11, *df *= 73, *p *≤ 0.038).

**Table 1:** Baseline sociodemographic, insight and neuropsychological variables comparison between patients remaining in the study and those who refused to participate in follow-up assessment.

	**Remaining patients (n = 56)**	**Refusers (n = 19)**	**t-pair test or chi-square**	**P**
**Age**	33,71 ± 9,00	35,00 ± 11,40	,50	,61
**Education **(years)	10,73 ± 3,24	9,21 ± 1,96	-2,44	,01
**N of episodes**	5,16 ± 4,74	6,00 ± 7,08	,57	,56
**Age at onset**	24,53 ± 6,80	24,73 ± 8,51	,10	,91
				
**Gender: males **(%)	35 (62,5)	24 (64.9%)	1.12	.57
**Civil: single **(%)	41 (73,2)	30 (81.1%)	3.15	.20
				
**PSYCHOPATHOLOGICAL DIMENSIONS**				
Psychotic	1 ± 1,18	1.37 ± 1.33	-1.13	.26
Negative	1,51 ± 1,15	1.58 ± 1.08	-.22	.82
Disorganization	,72 ± ,88	.70 ± .82	,08	.93
Depression	,50 ± ,73	.63 ± .83	-.65	.51
Mania	,23 ± ,50	.26 ± .45	-.24	.81
**INSIGHT MEASURES**^#^				
**ITAQ **(total score)	12,26 ± 7,58	10,63 ± 7,22	-,82	,41
**SUMD**				
Lack of Awareness, total score				
Current illness	2,66 ± 1,36	3,02 ± 1,30	,95	,34
Past illness	2,63 ± 1,22	2,79 ± 1,27	,49	,62
Mistaken Attribution, total score				
Current illness	2,65 ± 1,33	3,25 ± 1,29	1,53	,13
Past illness	2,53 ± 1,26	3,13 ± 1,10	1,76	,08
**AMDP**				
Lack of feeling of illness	1,42 ± 1,12	2,05 ± 1,07	2,11	,03
Lack of Insigt	1,64 ± 1,15	2,05 ± 1,07	1,36	,17
Refusal of treatment	,82 ± ,99	,78 ± 1,08	-,12	,90
**NEUROPSYCHOLOGICAL TESTS**				
Edinburgh test	12.47 ± .4.91	12.89 ± 8.16	-.44	.66
Information (WAIS)	11.04 ± .2.46	10.68 ± 2.19	.73	.46
Word Fluency	15.97 ± .4.64	15.63 ± 5.64	.37	.71
Stroop PC	30.53 ± .7.97	29.84 ± 9.30	.43	.66
Stroop Interference	42.41 ± .7.72	42.68 ± 10.14	-.18	.86
WISCONSIN CARD SORTING TEST				
Perseverative responses	30.81 ± .22.50	27.05 ± 17.04	.84	.40
Number of categories	3.88 ± .1.97	3.87 ± 2.01	-.04	.97
Trail Making test B (seconds)	181.96 ± 117.30	216.89 ± 149.66	-1.26	.21
Inmediate Verbal Memory	11.74 ± 3.81	10.92 ± 4.64	1.09	.27
Delayed VerbalMemory	11.72 ± 4.19	10.84 ± 4.39	1.06	.29

# Higher scores on ITAQ. Lower scores in awareness into symptoms and attribution scales of SUMD scale and AMDP items reflected better insight.

Differences between baseline and follow-up measures, after applying Bonferroni inequality correction to account for a high number of statistical procedures, are shown in Table 2. Neuropsychological performance was relatively stable across assessments, with the exception of improvement on Information (WAIS), the Interference task of the Stroop test and delayed verbal memory. Insight measures only showed significant improvement on the ITAQ score and a trend toward significant improvement on the AMDP 'Lack of feeling of illness' score.

**Table 2:** Differences between baseline and follow-up assessments on insight and neuropsychological variables.

	**BASELINE ASSESSMENT**		**FOLLOW-UP ASSESSMENT**		**STATISTICAL DIFFERENCES**	
	
	Mean	SD	Mean	SD	t-pair test	p
**Insight Measures# ITAQ **(total score)	12.26	7.58	16.21	7.61	-4.54	**.001**
**SUMD**						
Lack of Awareness, total score						
Current illness	2,66	1.36	2.45	1.15	1.69	.10
Past illness	2.63	1.22	2.39	1.20	1.02	.31
Mistaken Attribution, total score Current illness	2.65	1.33	2.97	1.12	-.79	.43
Past illness	2.53	1.26	2.83	1.28	-.26	.79
**AMDP**						
Lack of feeling of illness	1.42	1.12	1.01	1.14	3.12	.003
Lack of Insigt	1.64	1.15	1.30	1.08	2.30	.02
Refusal of treatment	.82	.99	.41	.83	2.46	.01

**Neuropsychological Tests**						
Information (WAIS)	11.04	2.46	11.87	2.54	-3.82	**.001**
Word Fluency	15.97	4.64	17.51	5.71	-2.06	.04
Stroop Interference	42.41	7.72	46.39	6.09	-4.12	**.001**
Perseverative Responses (WCST)	30.81	22.50	26.53	22.51	2.29	.02
Number of Categories (WCST)	3.88	1.97	4.06	2.03	-.72	.47
Trailb Making Test B (Seconds)	186.96	117.30	177.23	169.32	-.41	.68
Inmediate Verbal Memory	11.74	3.81	13.30	4.06	-2.69	.01
Delayed Verbal Memory	11.72	4.19	14.13	4.44	-3.94	**.001**

*Critical p value after Bonferroni correction (1 × 23) p ≤ 0.0021 (in bold)

# Higher scores on ITAQ. Lower scores in awareness into symptoms and attribution scales of SUMD scale and AMDP items reflected better insight.

Pearson correlation coefficients between cognitive and insight measures, both at baseline and at follow-up assessments, were carried out. As demonstrated in Table 3, no significant correlation coefficients were found after applying Bonferroni correction. The AMDP 'Refusal of treatment' score at follow-up showed trends towards a significant association with both the Interference task of Stroop test and the WCST 'number of categories' score.

**Table 3:** Pearson correlation coefficients between insight measures and neuropsychological test results at baseline (r_0_) and at follow-up assessments (r_1_)^ab^.

		**NEUROPSYCHOLOGICAL TESTS**															
		
		**7**		**8**		**9**		**10**		**11**		**12**		**13**		**14**	
		
		**r_0_**	**r_1_**	**r_0_**	**r_1_**	**r_0_**	**r_1_**	**r_0_**	**r_1_**	**r_0_**	**r_1_**	**r_0_**	**r_1_**	**r_0_**	**r_1_**	**r_0_**	**r_1_**
**ITAQ**	**1**	.03(56)	.02(56)	.04(56)	.12(56)	.04(56)	-.01(56)	.01(56)	-.03(56)	-.04(56)	-.06(56)	.06(56)	.03(56)	.07(56)	.16(56)	.10(56)	.23(56)
**SUMD scorings**	**2**	.10(47)	-.19(41)	.05(47)	-.13(41)	.07(47)	-.11(41)	-.10(47)	.06(41)	-.01(47)	-.01(41)	.08(47)	.21(41)	-.04(47)	-.15(41)	-.05(47)	-.10(41)
	**3**	.14(42)	.03(37)	-.11(42)	.05(37)	.08(42)	-.11(37)	.05(42)	.12(37)	-.09(42)	-.13(37)	-.05(42)	.17(37)	.03(42)	.05(42)	.01(42)	-.01(37)
**AMDP scorings**	**4**	.01(56)	.06(55)	.05(56)	.10(55)	-.12(56)	.12(55)	-.07(56)	.03(55)	.05(56)	-.10(55)	-.07(56)	-.06(55)	-.13(56)	.01(55)	-.03(56)	-.04(55)
	**5**	.12(56)	-13(55)	-.01(56)	-.10(55)	.10(56)	-.06(55)	.03(56)	14(55)	-.07(56)	-.10(55)	-.15(56)	.12(55)	-.03(56)	-.14(55)	-.06(56)	-.13(55)
	**6**	.04(56)	.02(55)	-.11(56)	-.08(55)	-.02(56)	.25(55)*	-.03(56)	-.09(55)	.07(56)	-30(55)**	-.16(56)	-.05(55)	-.08(56)	-.05(55)	-.06(56)	.03(55)

1. ITAQ; 2. Awareness of illness (current illness); 3. SUMD Mistaken attribution (current illness); 4. AMDP. Lack of feeling of illness; 5. AMDP. Lack of Insight; 6. AMDP Refusal of treatment; 7. Information (WAIS); 8. Word Fluency; 9. Stroop Interference; 10. WCST. Persev. Responses; 11. WCST Number of Categories; 12. Trailb Making B; 13. Inmediate Verbal Memory; 14. Delayed Verbal Memory.

a = In brackets, it is shown the number of patients in each correlation

b = Critical p value after Bonferroni correction (p ≤ .05) for two different correlation sets (6 × 8) was r = .48, p ≤ .001 (95% confidence interval for significant associations for n = 56 ranged from r = .24 to r = .66).

*: r = .25 p ≤ .05; ** r = .35 p ≤ .01

The factor analysis of the fourteen insight measures resulted in two insight dimensions, as described elsewhere [50]. The first factor was the 'general awareness' dimension with high loading on all SUMD items. The second factor reflected 'attitudes to treatment', with high weightings on the ITAQ total score and the AMDP refusal of treatment item and moderate loading on the AMDP lack of feeling ill and the AMDP lack of insight items.

A principal components factor analysis of the neuropsychological scores yielded five factors, which are named in order of extraction as follows: (a) 'general cognitive', (b) 'perseverative', (c) 'non-perseverative errors', (d) 'inefficient sorting', and (e) 'attentional-executive' factors. The following is a description of the results for the component symptoms and factor loading of symptoms on each factor. The 'general cognition' factor included the WAIS information test (.73), memory tests (immediate .90 and delayed .88), verbal fluency (.55), the Stroop color-word test (.56) and the Trail Making B test (-.54). The second factor, namely the 'perseverative' dimension, was comprised of the number of perseverative errors (.91) and the number of categories of the WCST (-.92). The third factor was the 'non-perseverative errors' dimension, which included non-perseverative errors (.70), unique responses (.77) and the number of perseverative errors (.53) on the WCST. The 'inefficient sorting' factor was made up of two WCST parameters: failure to maintain the set (-.93) and the number of corrects minus ten (-.86). The fifth factor was a mixed attentional and executive factor with high loading on the Stroop color-word test (.88) and the Trail Making B test (-.49). A five-factor solution was forcibly extracted at follow-up and an analogy between baseline and follow-up structures was established on the basis of composition and loading of items on factors. The order of extraction of factors varied from baseline to follow-up assessments.

The Bonferroni inequality correction was applied between insight and neuropsychological dimensions and no significant cross-sectional associations were found at baseline or follow-up (Table 4). The 'non-perseverative errors' factor of cognitive function at baseline showed a trend towards a significant correlation with the 'general awareness' dimension of insight (*r *= -.27). To account for differences in length of illness among patients, 'partial correlation' coefficient analyses between insight and neuropsychological dimensions, with illness duration as the covariate, were carried out and the same results were obtained. In addition, separate equation regressions were computed for insight dimensions at follow-up (dependent variable) with insight dimensions and each one of the five cognitive 'factor scores' at baseline (independent variables). Our results revealed that baseline cognitive dimensions did not predict change in insight dimensions at follow-up. Lastly, a similar profile of results of a lack of significant associations (i.e., between 'cognitive and insight' measures and dimensions) was found in the schizophrenia subset sample (*n *= 37). SPSS Regression output in the total sample is provided by the supplementary material to this article (see Additional file 1)

**Table 4:** Pearson correlation coefficients between insight and cognitive dimensions at baseline(r_0_) and at follow-up assessments (r_1_)^a^.

	INSIGHT DIMENSIONS	
	
	**General awareness dimension r_0_/r_1_**	**Attitudes to treatment dimension r_0_/r_1_**
**General cognitive factor**	-.02/.01	-.01/.01
**Perseverative factor**	.07/-.03	.05/-.07
**Non-perseverative errors factor**	-.27*/-.15	.10/-.10
**Inefficient sorting factor**	.01/-.15	.09/.10
**Attentional-executive factor**	.15/.07	.06/.02

p ≤ 0.05 **p ≤ 0.01

a Critical p ≤ .05 value after Bonferroni correction for a 5 × 2 matrix was r = .36 p ≤ .005 (in bold). *95% confidence interval for significant associations (n = 56) ranged from r = .10 to r = .56*.

## Discussion

Overall, our results support findings that insight dimensions and neuropsychological performance in patients suffering from psychoses are non-associated domains. Both cross-sectional and longitudinal analyses did not achieve the level for statistical association after the adequate statistical correction to avoid a Type I error. Moreover, a similar profile of 'lack of significant associations' was found in the subset of patients with schizophrenia.

The discrepancy between our results and those of some of the published studies might partly originate from several sources, such as the selection of different target samples, the use of unstandardized or non-specific scales for the assessment of insight, statistical shortcomings (i.e., lack of statistical correction to avoid Type I error), or unreliability in neuropsychological assessment. Heterogeneity across samples of previous studies was very common, since several studies comprised mixed samples [10,18,21,40,65] and others showed great differences in illness phase. As examples of the latter, samples of previous studies range from stable out-patients [7,8,38,39,41,66] to patients assessed after remission of acute episodes [9,48], relatively recent-onset patients [17,34] and first-episode psychotic patients [15,16,30,35] to rather chronic populations [22,31,37,47] or acute patients [26], and samples with non-selected patients to samples comprising 'high insight' patients or patients included in psychotherapeutic programs [19,45,46,67]. Some authors have suggested that cognitive deficits do not relate to insight in the earlier stages of the psychotic illness, but do in samples with longer mean duration of illness [38,47]. In regards to insight assessment, many studies have used unstandardized instruments, such as Item 12 on the Positive and Negative Symptom Scale (PANSS) [20,39,43,48,67] or insight items from general 'psychopathological inventories' (i.e., PSE [10,17] or AMDP [9,37]). These instruments have demonstrated limitations when attempting to capture the multidimensional aspects underlying insight.

Another source of inconsistency between studies emerged from statistical grounds. Most studies have analyzed their results using correlation or regression methods. However, an exception to this was the use of adequate statistical correction, such as the Bonferroni inequality [9,37] or one-tailed p [66], to avoid bias related to the performance of a high number of statistical procedures. For instance, after applying the Bonferroni correction to studies that reported significant correlations between insight and cognitive measures [18,20-26,31,32,42,46,67], few associations between insight or compliance and IQ remained at a statistically significant level [43]. In addition, only two studies by Young et al. [18,20] continued to show significant correlations between lack of insight and executive dysfunction. Caution is needed in the interpretation of our results supporting our original hypothesis, since negative results arose from the application of the Bonfferoni inequality correction to correlation coefficients. However, not only our results, but also the results of most studies that have rendered a similar profile of a lack of signification associations between insight and cognitive performance were analyzed with the Bonfferoni inequality correction.

There was some unreliability associated with neuropsychological assessment in studies that have focused on insight, such as limitations in the selection of neuropsychological tests and conceptual inconsistency. For instance, certain studies have exclusively used one test that is hypothetically related to these areas, such as the WCST [21,37,38,44] or tests only related to executive function [19,47,48,67] and then concluded that lack of insight was or was not associated with executive or frontal dysfunction. However, this selective approach to neuropsychological assessment cannot distinguish whether lack of insight is or is not associated with a global cognitive dysfunction [68]. Moreover, general tests for intelligence assessment [9,17,69], the use of only a single test [25,39] or the qualitative assessment of cognitive functioning through 'clinical vignettes' [32] are not appropriate neuropsychological instruments to use to reach firm conclusions regarding insight and cognitive functioning.

## Conclusion

Our present data is consistent with previous results from our group. We have previously hypothetized that insight dimensions are semi-independent domains of psychopathology regarding schizophrenic symptomatology. Insight dimensions have a 'primary' origin from a Bleulerian perspective [9,50,70]. In this regard, lack of insight should be considered a characteristic manifestation of patients suffering from psychosis. Our hypothesis is also supported by the lack of significant correlations between lack of insight and cognitive performance, which are found in other 'characteristic symptoms' of psychosis such as delusions or hallucinations. This does not mean that there is no biological substrate for psychotic symptoms, but that the relationships between clinical and cognitive domains seem to be too complex to be reduced to a single direct association.

There are a number of limitations in the present study that might reduce the strength of our results and should therefore be acknowledged. First, the use of factor scores to summarize insight measures might have resulted in different insight constructs compared to other studies. However, an inspection of the raw data led us to disregard any significant associations that were found between cognitive and insight measures. Second, missing values on the SUMD scale were replaced by its mean value and included in factor analysis conjointly with other insight measures. These other insight measures included values resulting from the ITAQ and AMDP instruments, which were available for all patients. This modification in the SUMD scale should be taken into account when comparing our results with other studies, since our methods used to extract factor scores might generate different insight constructs. Third, in spite of the fact that our sample was one of the largest in the literature, the inability to detect significant associations might represent a Type II error (i.e., lack of statistical power due to small sample size). In fact, for the highest correlation in Table 3 (correlation of .30 between AMDP refusal of treatment and WCST number of categories at follow-up), a sample size of 85 would have been needed to establish that correlation alone as statistically significant (power ≥ 0.80). Similarly, a sample size of 105 would have been needed to determine the highest correlation in Table 4 (correlation of -.27 between 'non-perseverative' factor and 'general awareness' dimension) as statistically significant. And fourth, cognitive assessment of patients during acute episodes might denote greater crises rather than poorer performance. However, the first point of assessment occurred once our patients were stabilized from the episode of psychosis, which was more than 3 weeks after the time of admission.

A pending question that can be derived from our results is: What are the neurobiological underpinnings of lack of insight? The absence of statistically significant associations between lack of insight and cognitive performance does not imply that insight dimensions do not hold pathophysiological correlates. On the contrary, it has been suggested that complex symptoms, such as lack of insight, are greatly influenced and distorted by the individual's socio-cultural background and language, as well as by the process of symptom formation itself. This occurs because less elaborate symptoms are more related to the biological substrate. In other words, the more complex the symptom, the farther the original signal is from the brain [71]. Following this line of reasoning, the search for 'clinical mediators' or simpler domains of psychopathology, such as hope [72], affect [73] and metacognition [74], might be relevant for understanding the neurobiological correlates of insight dimensions. Future studies that investigate the neurobiological dysfunction underlying psychosis should undertake the research starting with less-structured behaviors or from 'basic' dimensions from lower hierarchical levels [75].

## Abbreviations

SUMD: Scale to assess Unawareness of Mental Disorder. ITAQ: Insight and Treatment Attitudes Questionnaire. AMDP: Assessment and Documentation in Psychopathology. CASH: Comprehensive Assessment Schedule History. WCST: Wisconsin Card Sorting Test.

## Competing interests

The author(s) declare that they have no competing interest.

## Authors' contributions

MJC designed the study and participated as the coordinator. MJC and VP carried out the clinical and psychopathological assessments of patients. The assessment of insight was done by MJC, VP and AZ. Neuropsychological evaluations were carried out by AZ. Statistical analyses were performed by MJC, AZ and MZ. MJC and AZ drafted and updated the manuscript. All authors approved the final manuscript.

## Pre-publication history

The pre-publication history for this paper can be accessed here:



## Supplementary Material

Additional File 1 Regression output Biomedcentral Psychiatry. SPSS Regression output in the total sample. Click here for file
