# Supplementary material for: Insight dimensions and cognitive function in psychosis: a longitudinal study
Source: BMC Psychiatry. 2006 May 31;6:26. doi: 10.1186/1471-244X-6-26 (PMC1489928; doi:10.1186/1471-244X-6-26)
Supplement: Additional File 1 — Regression output Biomedcentral Psychiatry. SPSS Regression output in the total sample. [file 1471-244X-6-26-S1.doc]

**BASELINE COGNITIVE FACTORS:**

**COGNI01**

**COGNI02**

**COGNI03**

**COGNI04**

**COGNI05**

**DIFFERENCES BETWEEN BASELINE AND FOLLOW-UP ON INSIGHT FACTOR1= AVERDIF1**

**DIFFERENCES BETWEEN BASELINE AND FOLLOW-UP ON INSIGHT FACTOR1= AVERDIF2**

**SPSS REGRESSION OUTPUT**

**(Stepwise and Enter methods)**

REGRESSION

/MISSING LISTWISE

/STATISTICS COEFF OUTS R ANOVA

/CRITERIA=PIN(.05) POUT(.10)

/NOORIGIN

/DEPENDENT cogni01

/METHOD=STEPWISE averdif1 averdif2.

**Regresión**

REGRESSION

/MISSING LISTWISE

/STATISTICS COEFF OUTS R ANOVA

/CRITERIA=PIN(.05) POUT(.10)

/NOORIGIN

/DEPENDENT cogni02

/METHOD=STEPWISE averdif1 averdif2.

**Regresión**

REGRESSION

/MISSING LISTWISE

/STATISTICS COEFF OUTS R ANOVA

/CRITERIA=PIN(.05) POUT(.10)

/NOORIGIN

/DEPENDENT cogni03

/METHOD=STEPWISE averdif1 averdif2.

**Regresión**

REGRESSION

/MISSING LISTWISE

/STATISTICS COEFF OUTS R ANOVA

/CRITERIA=PIN(.05) POUT(.10)

/NOORIGIN

/DEPENDENT cogni04

/METHOD=STEPWISE averdif1 averdif2.

**Regresión**

REGRESSION

/MISSING LISTWISE

/STATISTICS COEFF OUTS R ANOVA

/CRITERIA=PIN(.05) POUT(.10)

/NOORIGIN

/DEPENDENT cogni05

/METHOD=STEPWISE averdif1 averdif2.

**Regresión**

REGRESSION

/MISSING LISTWISE

/STATISTICS COEFF OUTS R ANOVA

/CRITERIA=PIN(.05) POUT(.10)

/NOORIGIN

/DEPENDENT cogni01

/METHOD=enter averdif1 averdif2.

**Regresión**

REGRESSION

/MISSING LISTWISE

/STATISTICS COEFF OUTS R ANOVA

/CRITERIA=PIN(.05) POUT(.10)

/NOORIGIN

/DEPENDENT cogni02

/METHOD=enter averdif1 averdif2.

**Regresión**

REGRESSION

/MISSING LISTWISE

/STATISTICS COEFF OUTS R ANOVA

/CRITERIA=PIN(.05) POUT(.10)

/NOORIGIN

/DEPENDENT cogni03

/METHOD=enter averdif1 averdif2.

**Regresión**

REGRESSION

/MISSING LISTWISE

/STATISTICS COEFF OUTS R ANOVA

/CRITERIA=PIN(.05) POUT(.10)

/NOORIGIN

/DEPENDENT cogni04

/METHOD=enter averdif1 averdif2.

**Regresión**

REGRESSION

/MISSING LISTWISE

/STATISTICS COEFF OUTS R ANOVA

/CRITERIA=PIN(.05) POUT(.10)

/NOORIGIN

/DEPENDENT cogni05

/METHOD=enter averdif1 averdif2.

**Regresión**
